# Cost estimation alongside a multi-regional, multi-country randomized trial of antenatal ultrasound in five low-and-middle-income countries

**DOI:** 10.1186/s12889-021-10750-8

**Published:** 2021-05-20

**Authors:** B. W. Bresnahan, E. Vodicka, J. B. Babigumira, A. M. Malik, F. Yego, A. Lokangaka, B. M. Chitah, Z. Bauer, H. Chavez, J. L. Moore, L. P. Garrison, J. O. Swanson, D. Swanson, E. M. McClure, R. L. Goldenberg, F. Esamai, A. L. Garces, E. Chomba, S. Saleem, A. Tshefu, C. L. Bose, M. Bauserman, W. Carlo, S. Bucher, E. A. Liechty, R. O. Nathan

**Affiliations:** 1 Department of Radiology, University of Washington, Seattle, WA USA; 2 Department of Pharmacy, University of Washington, Seattle, WA USA; 3 Department of Global Health, University of Washington, Seattle, WA USA; 4 Aga Khan University, Karachi, Pakistan; 5 Moi University, Eldoret, Kenya; 6 Kinshasa School of Public Health, Kinshasa, Democratic Republic of the Congo; 7 University of Zambia, Lusaka, Zambia; 8 University of Francisco Marroquin, Guatemala City, Guatemala; 9 RTI International, Durham, NC USA; 10 Columbia University, New York, NY USA; 11 INCAP, Guatemala City, Guatemala; 12 University of North Carolina, Chapel Hill, NC USA; 13 University of Alabama Birmingham, Birmingham, AL USA; 14 Indiana University, Indianapolis, IN USA

**Keywords:** Maternal health, Antenatal care, Delivery, Cost, Health economics, Low-and-middle-income countries

## Abstract

**Background:**

Improving maternal health has been a primary goal of international health agencies for many years, with the aim of reducing maternal and child deaths and improving access to antenatal care (ANC) services, particularly in low-and-middle-income countries (LMICs). Health interventions with these aims have received more attention from a clinical effectiveness perspective than for cost impact and economic efficiency.

**Methods:**

We collected data on resource use and costs as part of a large, multi-country study assessing the use of routine antenatal screening ultrasound (US) with the aim of considering the implications for economic efficiency. We assessed typical antenatal outpatient and hospital-based (facility) care for pregnant women, in general, with selective complication-related data collection in women participating in a large maternal health registry and clinical trial in five LMICs. We estimated average costs from a facility/health system perspective for outpatient and inpatient services. We converted all country-level currency cost estimates to 2015 United States dollars (USD). We compared average costs across countries for ANC visits, deliveries, higher-risk pregnancies, and complications, and conducted sensitivity analyses.

**Results:**

Our study included sites in five countries representing different regions. Overall, the relative cost of individual ANC and delivery-related healthcare use was consistent among countries, generally corresponding to country-specific income levels. ANC outpatient visit cost estimates per patient among countries ranged from 15 to 30 USD, based on average counts for visits with and without US. Estimates for antenatal screening US visits were more costly than non-US visits. Costs associated with higher-risk pregnancies were influenced by rates of hospital delivery by cesarean section (mean per person delivery cost estimate range: 25–65 USD).

**Conclusions:**

Despite substantial differences among countries in infrastructures and health system capacity, there were similarities in resource allocation, delivery location, and country-level challenges. Overall, there was no clear suggestion that adding antenatal screening US would result in either major cost savings or major cost increases. However, antenatal screening US would have higher training and maintenance costs. Given the lack of clinical effectiveness evidence and greater resource constraints of LMICs, it is unlikely that introducing antenatal screening US would be economically efficient in these settings--on the demand side (i.e., patients) or supply side (i.e., healthcare providers).

**Trial registration:**

Trial number: NCT01990625 (First posted: November 21, 2013 on https://clinicaltrials.gov).

**Supplementary Information:**

The online version contains supplementary material available at 10.1186/s12889-021-10750-8.

## Background

According to the World Health Organization, approximately 95% of maternal and 98% of neonatal deaths occur in low- and middle-income countries (LMICs). Global estimates from 2017 indicate that more than 800 women die each day due to preventable causes related to pregnancy and childbirth [1]. Despite improvements in maternal and child mortality in LMICs during the past decades resulting from strategic investments and initiatives by countries, health organizations, and donors, there is limited evidence consistently demonstrating the clinical effectiveness of many interventions [2]. Assessments of the economic impact of implementing innovative strategies to improve health outcomes in LMICs have received increased attention, but data gaps and other challenges persist [3, 4].

Diagnostic tests present a compelling case for studying the potential costs and consequences in LMICs of technologies used regularly in higher-income countries. One challenge, however, of assessing the value of multi-use diagnostic tests is that the same technology may have several indications with differing value profiles associated with each type of use. Ultrasound (US), for example, may be used to make hospital-based diagnoses, and it also has been used as a screening tool for many years during antenatal care (ANC) to identify high-risk pregnancies, the presence of potential complications, and to determine gestational age [5–7]. In LMICs, the use of antenatal screening US was thought to potentially add value by encouraging earlier ANC attendance, allowing for earlier identification of high-risk pregnancies, and helping mothers appropriately plan their ANC and delivery based on their risk profile [8]. Our current health economic study focuses only on the usage for routine antenatal screening ultrasound, not on all uses of ultrasound.

Innovations in US technologies have reduced equipment size, improving portability and making point-of-care US more feasible, resulting also in lower US equipment acquisition costs over time. However, questions remain related to the appropriate, effective, and efficient use of US for specific indications. Prior to making large-scale investments in the adoption and diffusion of portable US by global stakeholders, decision makers can benefit from having more clinical and economic evidence. The First Look cluster-randomized controlled trial (CRCT) in five diverse LMICs generated evidence about the clinical effectiveness of portable, community-based, antenatal screening US by evaluating the impact on maternal mortality, maternal near-miss, stillbirth, and neonatal mortality [9, 10]. Given the “intermediate outcomes” characteristic of screening or diagnostic tests, including for ultrasound, it was important that our CRCT and accompanying economic study also tracked non-ultrasound antenatal care visits, patient referrals, deliveries, and hospital-related events [11].

The current paper reports resource use and cost data collected alongside this CRCT as part of assessing potential economic efficiency gains of introducing antenatal screening US in five LMICs: Democratic Republic of Congo (DRC), Guatemala, Kenya, Pakistan, and Zambia. Our primary objective was to estimate average patient-level costs for facilities in the participating countries for portable antenatal screening US use, general ANC, and delivery events based on health facility location and type of delivery. The overall trial results have been reported and suggested that, in general, the introduction of routine antenatal screening US is unlikely to be more efficacious compared to standard of care [12]. Our cost estimation focused on aggregated estimates for ANC-related use, delivery-related use, and complications for combined standard of care and experimental intervention groups in each country, due to the similar effectiveness and process outcomes found between arms in the CRCT, implying similar event costs between groups. Despite our original goal to assess costs in relation to effectiveness difference between groups, we believe these data are still useful for others doing research related to ANC or maternal health use and costs, or developing policies on antenatal interventions in LMIC settings. We describe the methods for generating our estimates, present cost estimates for the five countries, and discuss potential implications for our findings for research practice and policy.

## Methods

### Overview and aims

The primary objective of this economic study performed alongside the First Look CRCT was to estimate average patient-level costs in participating countries comparing the US group to standard of care. We collected resource use for ANC visits and delivery-related events based on health facility location and type of delivery (cesarean section or vaginal birth). In addition, we wanted to estimate facility-based costs associated with complications experienced during pregnancy, at the time of delivery, and during a six-week post-partum period. Our approach was to establish unit costs for particular resource use, and to construct key event average costs from these unit cost estimates. We developed these cost estimates through site visits in collaboration with local health researchers, thus providing a clinic or hospital facility perspective for our use and cost assessment. We examined relative prices within countries for different resources and events, and converted them to 2015 United States dollars (USD) for general comparisons across countries, despite differences in health systems in participating countries.

### Population

Our study population included pregnant women enrolled in the CRTC intervention clusters and women in control (standard of care) clusters participating in the Global Network Maternal Newborn Health Registry (GN MNHR) in five countries: the Democratic Republic of Congo (DRC), Guatemala, Kenya, Pakistan, and Zambia [13]. Each cluster was a defined geographic area generally served by a single health center and its catchment area with roughly 500 births per year. Further details about the CRCT study population and study design are detailed elsewhere [12]. The CRCT adhered to CONSORT guidelines and the CONSORT diagram is presented in Supplemental Figure 1. The overall sample included normal (low-risk) pregnancies and high-risk pregnancies identified as multiple gestation, placenta previa, and breech presentation. Each participant provided written informed consent prior to study participation, including for the use of their study data.

### Data sources

We used three primary data sources to inform our economic assessment. First, for intervention clusters, we used the CRCT data on the number of ANC visits, delivery location, type of delivery (vaginal or cesarean section), and select pregnancy-related complications collected during a portion of the trial. Second, similar data for the control-group clusters were obtained from the GN MNHR [13]. Third, we collected average time and cost estimates for typical ANC, delivery, and complication-related interventions or events using consistent, standard data collection tools in each country, with local health experts interviewing hospital administrators and using reimbursement schedules as proxies for costs when available. Due to similar outcomes observed in the CRCT [12], we report country-level averages for ANC and delivery-related resource use for all intervention and control patients combined within each country for the average probability parameters of visit-related or facility-related events.

Our cost estimates focused on public health facilities in participating countries, at the local health clinic level and the hospital (facility) level. We also included private facility data collection in Pakistan, DRC, and Kenya to provide a range of estimates. Data collection in Zambia included several public facilities, most often with similar fee structures. We obtained data from one representative rural public hospital in Guatemala.

### Perspective of cost assessment

The perspective for the cost assessment was a health facility perspective and this can be viewed as a health delivery-system perspective, accounting for visits, deliveries, and complication events. We focused on facility-related reimbursement for services, despite the fact that some countries do not have public or private health insurance provision for our study participants for ANC or delivery services, implying mothers or families incur the financial burden rather than via payer reimbursement.

### Time horizon of resource use

The period of our data collection included visits or complications during the ANC pre-delivery period, the delivery event, and a 6-week period post-delivery during which complications were managed in a hospital setting.

### Types of average cost data collected

We collected data on average gross-level costs for typical ANC resource use and delivery-related events, as well as low and high ranges for each type of service. We used per patient average cost estimates to calculate ANC and in-hospital costs for patient care. Our data tools included the collection of inpatient and outpatient ANC costs, as well as costs for delivery-related events and pregnancy-related complication event care. For the cost estimation, we did not have data for specific CRCT patient events in the hospital or assess patient-related clinic or hospital records such as for hospital stays related to pregnancy complications or delivery events. All costs were reported in local currencies and converted to 2015 USD for comparisons [14]. We converted average cost estimates to 2015 USD with exchange rates listed in Table 1 [15–19]. We did not calculate costs for US equipment, training sonographers, or maintaining equipment/training.

**Table 1:** Select population-level statistics for countries participating in the trial (2015)

2015 rates/statistics	DRC	Kenya	Zambia	Guatemala^c^	Pakistan
Population (1000’s)	77,267	46,050	16,212	14,389	188,925
Per capita income (USD 2015)	497^b^	1337^b^	1332^b^	3924^b^	1357^b^
Currency exchange rate per 1 USD (June 2015 USD)	921.29	99.19	7.55	7.63	101.82
Total live births (1000’s)	3217	1571	645	467	5451
Maternal mortality ratio^a^ (per 100 live births)	0.6930	0.5100	0.2240	0.1100	0.1780
Neonatal mortality rate^b^ (per 100 live births)	3	2.2	2.1	1.5	4.6
Calculated ratio of neonatal mortality to maternal mortality (per 100 live births)	4.33	4.31	9.38	13.64	25.84

Notes: ^a^Converted to per 100 live births from per 100,000,

^b^Converted to per 100 live births from per 1000

^c^Latest available mortality statistics for Guatemala were from 2010

### Estimating antenatal care resource use, other resource use, and costs in countries

We calculated the average amount of resources used associated with ANC visits (with and without antenatal screening US), procedures, or interventions including outpatient and inpatient services, hospital stays and in-facility delivery procedures, and resource use based on reported complications in a portion of study participants (trial and registry). Cost and probability estimates for standard ANC services and complication-related services performed in facility settings for maternal and neonatal complications were used to estimate resource use and event costs. Although antenatal screening US use was primarily based on use in one randomized cohort in the CRCT (i.e., intervention group), we provide an overall estimate of ANC costs based on the average number of antenatal screening US visits recorded for patients in the trial. We calculated a combined ANC visit cost estimate that included non-US visits and antenatal screening US visits recorded for each country-level sample from the CRCT, subtracting the estimated number of US visits from the overall number of ANC visits to avoid double counting.

For ANC visits, the average number of visits reported in countries was multiplied by the outpatient facility cost of an ANC visit, for visits without US and for visits including antenatal screening US. The average number of ANC visits calculation used data from the GN MNHR and the CRCT. The average number of overall ANC visits in countries was calculated net of the proportion of patients with one, two, three, or four or more US visits using reported visits from the CRCT. A small percentage of persons (all countries < 1%) with more than four visits with antenatal screening US were assumed to have four US visits for ANC cost calculations.

Our facility-level “gross costing” reimbursement-based approach used reported average cost data for each type of visit or other service provided in a health facility, or outpatient setting for ANC visits. Although country-level data collection included estimates from several facilities, available services differed at specific facilities within countries, resulting in some zero dollar cost estimates for services when they were not available in a particular facility. For country-level average cost estimates, only cost estimates greater than zero were used for base-case estimates when a resource item was available at facilities in countries. Some services in countries were bundled as part of overall delivery-related episode-based reimbursement and thus did not have item-level cost estimates.

Probabilities for delivery location were obtained from the CRCT and GN MNHR data, indicating whether deliveries occurred at one of three settings: local/village, non-hospital clinic, or a hospital/health center. Average costs for deliveries based on delivery location were estimated using the unit cost estimate for services and the proportion of mothers reported to have delivered using cesarean section. We used CRCT and GN MNHR data for cesarean delivery rates and assumed cesarean deliveries occurred only in the hospital facility for costing purposes. For these deliveries we also used country-level unit costs for hospital stays associated with cesarean deliveries. We did not assume hospital stay costs for non-cesarean deliveries. Delivery costs for a subset of mothers identified with higher-risk pregnancies are also reported. Countries with public reimbursement mechanisms, e.g., Kenya and Zambia, reported “lump sum” estimates for deliveries in public facilities which included payment (cost) for services associated with addressing birth-related complications in mothers and children. Since we did not have a facility unit cost estimate for local-level or clinic-level deliveries, base case estimates assumed a proportion of the hospital-based delivery cost for a non-cesarean delivery, at a 25% rate for local/village deliveries, and 50% for non-hospital clinic deliveries. Base-case estimates used reported unit cost averages and a range for cesarean delivery costs for the hospital services.

The use of select hospital/referral services for treatment of complications was reported in a sub-sample of the CRCT for maternal complications in the GN MNHR for neonatal complications, along with major services used. Since we expected hospital-based management for complications to be a higher-cost item, we incorporated pregnancy-related complication collection at sites, to complement similar GN MNHR data on complications. We used these reported proportions of maternal and neonatal complications and applied unit cost estimates to each type of service to estimate average facility-based management costs for these types of typical complications. Maternal health complication management included treatment with antibiotics, hysterectomy or other surgery, use of anesthesia or blood transfusion, and whether inpatient US or chest radiograph was used. Select neonatal services use included hospitalizations, neonatal bath, antibiotics, oxygen, mechanical ventilator, medical eye or cord care, and vitamin K treatment. Unit prices for services were multiplied times the proportion of patients in the CRCT or GN MNHR reporting use of these services specifically for pregnancy-related complications. Countries with public reimbursement mechanisms, e.g., Kenya and Zambia, reported “lump sum” estimates for deliveries in public facilities which represented full reimbursement (cost) for services associated with managing complications in mothers and children. Thus select resource use items in countries are indicated as zero USD cost estimates. In some cases zero USD unit costs indicate the lowest-resourced rural hospitals sampled did not have all services/equipment available, such as in DRC.

### Sensitivity testing of specific parameters

Sensitivity analysis was conducted for select ANC visit costs and delivery costs for countries. ANC visits with and without antenatal screening US were primary variables of interest and the low and high ranges of these outpatient items were used in testing the impact on ANC visit-related average costs, using the average number of each type of visit in our country-level cohorts. For each country, we first used the low range cost estimates of ANC visits with and without US as well as the high ranges of each type of visit (two-way sensitivity). We then used the low and high range of either ANC with or without US and the base-case cost estimate of the other type of visit (one-way sensitivity analysis). Likewise, low and high ranges of delivery cost variables were tested in sensitivity analysis, for both cesarean and non-cesarean modes of delivery. In addition, since our data collection focused on hospital facility-level cost estimation, we did not have cost estimates for home- or village-level deliveries or clinic-level deliveries. Therefore, we adjusted our proportional cost assumptions for home/village deliveries and clinic-level deliveries from 25 and 50% (base case) of hospital-based non-cesarean deliveries, respectively, and tested 10 and 35% of hospital costs for home−/village-level and 25 and 60% for clinic-level. To provide a high-end cost range for cesarean section deliveries with complications, sensitivity analyses were performed by incorporating additional hospital stay cost estimates associated with 100% of cesarean deliveries, in addition to the standard average costs for cesarean section deliveries.

## Results

Table 1 presents several population-level demographic statistics for participating countries to provide context for understanding variability in estimates among our sample. Per capita Gross Domestic Product (GDP), a measure of national income, varied substantially among participating countries in 2015 (497 USD in DRC, 1337 USD in Kenya, 1332 USD in Zambia, and 3924 USD in Guatemala, 1357 USD in Pakistan [14]. Population size and live births per 1000 in the population also varied by several fold, as well as maternal and neonatal mortality rates per 100 live births.

### Average cost estimates for outpatient clinic and facility-based antenatal care and delivery care

Average per mother antenatal service use cost estimates and ranges are presented in Table 2. Average item-level costs of resources or services, i.e., reimbursement amounts as proxies, generally varied in alignment with income levels in countries, but not for all services. For example, average cost estimates in countries for ANC outpatient visits without antenatal screening US were similar and ranged from 3─6 USD in countries. Outpatient antenatal visits including antenatal screening US were more variable in cost and estimated to be 4 USD in Pakistan and Guatemala, while estimates ranged from 10─13 USD in DRC, Kenya, and Zambia. Low and high ranges of estimates are also presented in Table 2 and were used in sensitivity analyses. Select unit cost estimates in the DRC, for example, such as for anesthesia use, likely represent a high-end cost for services which may not be available in rural health facilities or may not be reimbursed at that level. Lower antenatal screening US costs in Pakistan and Guatemala are likely related to US being more available in their healthcare systems.

**Table 2:** Facility-related unit cost estimates for antenatal care services in countries (2015 USD)

	Dem. Rep. of Congo			Kenya			Zambia			Guatemala			Pakistan		
	Average	Low	High	Average	Low	High	Average	Low	High	Average	Low	High	Average	Low	High
**Facility Costs**															
Outpatient ANC Visit	4.70	0.54	16.28	4.41	1.01	10.08	5.74	3.97	9.27	3.28	2.88	3.67	3.72	1.47	6.87
Inpatient ANC Visit	3.80	3.80	3.80	12.78	5.04	40.33	7.87	6.62	9.27	3.15	2.62	3.54	3.72	1.47	6.87
Outpatient Ultrasound Visit	12.83	2.00	30.00	10.33	8.07	15.12	13.25	6.62	19.87	4.19	3.41	4.98	4.30	2.46	5.89
Inpatient Ultrasound Visit	16.28	16.28	16.28	10.64	8.07	15.12	13.25	6.62	19.87	5.50	4.46	6.55	4.30	2.46	5.89
Consultation with a Specialist, separate from ultrasound	9.77	9.77	9.77	4.44	0.20	10.08	16.56	13.25	19.87	4.33	3.54	4.98	19.08	1.37	52.91
Hospital Overnight Stay	2.97	1.58	5.43	4.11	1.01	9.07	23.18	19.87	26.49	17.04	15.07	19.13	14.35	4.91	23.41
ICU Overnight Stay	12.10	1.58	30.39	0.00	0.00	0.00	46.36	39.74	52.98	69.72	46.13	93.32	141.27	98.21	176.78
Delivery in hospital with cesarean section	108.50	10.85	287.64	156.27	50.41	272.20	99.34	79.47	119.21	44.46	39.15	49.80	202.85	116.87	294.64
Delivery in hospital without cesarean section	41.68	5.43	75.98	55.20	19.16	100.82	90.32	56.29	105.96	26.02	24.71	27.33	75.88	53.21	127.68
Neonatal Resuscitation with Bag and Mask	N/A	0.00	0.00	N/A	0.00	0.00	3.31	1.59	6.62	1.57	1.31	1.83	4.66	4.39	4.92
Neonatal Bath	N/A	0.00	0.00	N/A	0.00	0.00	1.32	0.66	1.99	1.31	1.18	1.31	N/A	0.00	0.00
Neonatal Provision of Antibiotics	7.00	5.00	30.00	7.06	5.04	9.07	1.32	0.66	1.99	8.26	8.13	8.26	6.53	3.38	14.73
Neonatal Oxygen	29.31	29.31	29.31	1.01	1.01	1.01	1.32	0.66	1.99	6.16	5.11	7.21	4.66	4.39	4.92
Neonatal Mechanical Ventilation	N/A	0.00	0.00	N/A	0.00	0.00	1.32	0.66	1.99	39.58	32.77	46.40	4.66	4.39	4.92
Neonatal Medicinal Eye Care	0.71	0.54	0.87	0.20	0.20	0.20	1.32	0.66	1.99	4.72	4.33	5.11	N/A	0.00	0.00
Neonatal Medicinal Cord Care	N/A	0.00	0.00	N/A	0.00	0.00	1.21	0.13	1.99	0.66	0.52	0.79	N/A	0.00	0.00
Neonatal Provision of Vitamin K	1.90	0.54	3.26	2.02	2.02	2.02	0.74	0.13	1.99	1.31	1.31	1.44	0.58	0.58	0.58
Neonatal Chest X-Ray for Pneumonia	13.76	12.00	16.28	8.07	8.07	8.07	5.30	2.65	7.95	9.70	9.04	10.22	2.50	2.50	2.50
Neonatal hospitalization	5.43	5.43	5.43	2.52	2.02	3.02	33.11	26.49	39.74	79.95	77.98	81.91	73.57	73.57	73.57
Hospital Stay for C-Section	27.14	10.85	54.27	38.81	5.04	90.73	33.11	26.49	39.74	155.96	77.98	233.94	207.23	116.87	294.64
Treatment with Antibiotics	7.00	5.00	30.00	36.97	10.08	70.57	3.31	1.99	4.64	12.00	8.00	16.00	9.58	5.89	14.73
Hospital Stay for Hysterectomy	27.14	10.85	54.27	214.24	50.41	302.45	26.49	19.87	33.11	155.96	77.98	233.94	171.87	108.03	294.64
Hospital Stay for Other Surgery	32.56	10.85	54.27	214.24	50.41	302.45	90.88	19.87	132.45	155.96	77.98	233.94	82.50	82.50	82.50
Use of Anesthesia	143.82	143.82	143.82	10.08	10.08	10.08	N/A	0.00	0.00	20.58	17.96	23.20	N/A	0.00	0.00
Blood transfusion (cost per pint)	9.95	12.48	12.48	11.76	0.50	20.16	N/A	0.00	0.00	47.84	30.14	65.53	19.48	11.79	29.46

Note: N/A indicates service or resource not available or item is bundled with reimbursement for delivery-based care

Note: Antibiotic prices for Guatemala estimated from external source, other surgery has same cost as hysterectomy

For Kenya, other surgery costs assumed same as for hysterectomy

Additional estimates presented in Table 2 include cost ranges for standard ANC resource use including hospital stays, deliveries in hospital facilities with and without cesarean section and estimates for managing complication events. Hospital overnight stays per night ranged from a low of 3 USD in the DRC to a high-end average cost estimate of 23 USD in Zambia (Kenya 4 USD, Guatemala 17 USD, and Pakistan 14 USD). Costs for cesarean deliveries in all countries were higher than vaginal deliveries, with cesarean deliveries being roughly twice as costly or more.

Table 3 presents probabilities for the location of deliveries and maternal and neonatal events that were captured and used to calculate costs deliveries and complication events presented in Tables 4 and 5. Hospital deliveries and cesarean deliveries were highest in Guatemala, and lowest in DRC. Estimates in Table 4 account for scenarios with non-US visits and antenatal screening US visits, deliveries based on recorded location of child delivery (home/village, non-hospital clinic, and hospital), and whether hospital deliveries used cesarean section or non-cesarean methods.

**Table 3:** Event probabilities for delivery settings and maternal and neonatal events related to complications

	DRC	Kenya	Zambia	Guatemala	Pakistan
Location of child delivery and probability of Cesarean section delivery					
**Delivery location and probability (N for overall sample)**^a^	4818	9170	9367	16,287	7243
**Overall sample average**					
Local village or home setting (%)	19.51	26.52	18.04	42.11	37.48
Non-hospital clinic setting (%)	66.15	54.61	58.13	0.86	29.30
Hospital setting (%)	14.34	18.87	23.83	57.03	33.22
Proportion of deliveries with Cesarean section delivery (all trial participants) (%)	1.12	2.26	1.20	27.78	11.46
**Higher-risk (complicated pregnancy) sub-sample of overall sample (n)**	953	1143	1759	5458	2367
Estimated proportion of higher-risk pregnancies (% of total sample per country)	19.78	12.46	18.77	33.51	33.00
**Delivery location and probability**					
Local village or home setting (%)	18.47	21.00	14.10	37.71	35.02
Non-hospital clinic setting (%)	62.33	52.06	52.25	0.64	25.64
Hospital setting (%)	19.20	26.95	33.66	61.65	39.33
**Type of delivery**					
Proportion of deliveries with Cesarean section delivery (%)	3.67	7.87	3.47	34.15	14.91
**Maternal health-related complication probabilities**^a^					
*Maternal healthcare resource use (N)*	145	142	163	1282	147
Antibiotics (% yes)	86.25	78.70	43.95	83.30	93.90
Hysterectomy after complication (% yes)	2.85	0.65	0.00	0.90	0.00
Other surgery (% yes)	2.85	2.75	0.45	11.30	2.05
Anesthesia (% yes)	17.50	30.35	8.40	61.35	2.75
Blood transfusion (% yes)	13.25	6.10	3.20	4.50	21.70
Inpatient ultrasound (% yes)	9.50	11.65	8.20	78.20	27.90
Chest radiograph (% yes)	2.00	0.00	0.95	1.80	1.40
**Neonatal health-related complication probabilities**^b^					
Select neonatal healthcare resource use (sample ranges in countries per use type, N)^c^	4660-4908	7493-9288	6363-9456	15,827-16,394	3544-7284
Neonatal hospitalization (% yes)	0.15	0.75	0.50	3.35	2.40
Neonatal bath within six hours after delivery (% yes)	0.10	27.85	0.80	36.80	13.30
Neonatal antibiotics (% yes)	1.55	0.50	0.20	3.80	10.80
Neonatal oxygen use (% yes)	0.00	0.80	0.35	4.30	12.05
Mechanical ventilation (% yes)	0.40	0.45	0.15	0.55	0.20
Medical eye care (% yes)	1.25	15.20	1.00	56.25	0.90
Medicinal cord care (% yes)	2.65	2.00	1.40	99.30	2.30
Vitamin K (% Yes)	0.10	1.15	0.45	56.85	3.40

^a^Maternal health-related complications reported from sub-sample direct data collection from clinical trial

^b^Neonatal health-related complications reported from Maternal Health Registry

^c^Sample size range for specific neonatal complications included some variability

**Table 4:** Simplified country-level use and cost estimates of antenatal care services and delivery by setting

	DRC	Kenya	Zambia	Guatemala	Pakistan
**Antenatal care (ANC) visits (N for overall sample)**	4819	9177	9369	16,288	7251
Weighted-mean number of ANC visits overall, per person (#)	3.25	3.60	3.20	4.45	3.90
Mean per person cost for average number of ANC visits with no ultrasound (using mean # of ANC visits for overall sample) (2015 USD)	**15.28**	**15.87**	**18.37**	**14.58**	**14.53**
Overall number of visits (exams) with ultrasound (sub-sample of patients receiving at least one ultrasound) (n)	2291	3380	4408	7935	2404
Estimated percent of per person ultrasound visits (% for each)					
1	24.31	50.36	40.86	17.10	35.19
2	65.26	46.54	55.51	69.32	61.06
3	9.56	2.84	3.43	12.70	3.45
> = 4^c^	0.87	0.27	0.20	0.88	0.29
Weighted mean for number of ultrasound visits (of those having > = 1) (#)	1.87	1.53	1.63	1.97	1.69
Weighted-mean cost estimate for weighted-mean number of ANC visits with ultrasound (assumed outpatient) (2015 USD)	**24.00**	**15.81**	**21.59**	**8.28**	**7.25**
Calculated weighted-mean cost of overall ANC visits (average number of overall ANC visits net of estimated ANC visits with ultrasound to avoid double counting) (2015 USD)	**30.48**	**24.93**	**30.60**	**16.40**	**15.49**
**Delivery location and probability (N for overall sample)**^a^	4818	9170	9367	16,287	7243
**Overall sample average (% for each)**					
Local village or home setting (%)	19.51	26.52	18.04	42.11	37.48
Non-hospital clinic setting (%)	66.15	54.61	58.13	0.86	29.30
Hospital setting (%)					
Hospital delivery with cesarean section (%)	1.12	2.26	1.20	27.78	11.46
Hospital delivery without cesarean section (%)	13.22	16.61	22.63	29.26	21.76
**Cost of delivery by type**^d^ **(2015 USD)**					
Average per person cost of deliveries with cesarean section delivery (2015 USD)	108.50	156.27	99.34	44.46	202.85
Average per person cost, non-cesarean deliveries (2015 USD)	41.68	55.20	90.32	26.02	75.88
Calculated weighted-mean cost per mother estimate for deliveries for overall sample (non-Cesarean and Cesarean-Section; includes non-high-risk pregnancies and high-risk pregnancies)^b^ (2015 USD)	**22.54**	**31.43**	**51.96**	**22.81**	**57.98**
**Higher-risk (complicated pregnancy) sub-sample of overall sample (n)**	953	1143	1759	5458	2367
Estimated proportion of higher-risk pregnancies (% of total sample per country)	19.78	12.46	18.77	33.51	33.00
**Delivery location and probability**					
Local village or home setting (%)	18.47	21.00	14.10	37.71	35.02
Non-hospital clinic setting (%)	62.33	52.06	52.25	0.64	25.64
Hospital setting (%)					
Hospital delivery with cesarean section (%)	3.67	7.87	3.47	34.15	14.91
Hospital delivery without cesarean section (%)	15.53	19.07	30.19	27.50	24.42
**Cost of delivery by type**^d^ **(2015 USD)**					
Average per person cost of deliveries with cesarean section delivery (2015 USD)	108.50	156.27	99.34	44.46	202.85
Average per person cost, non-cesarean deliveries (2015 USD)	41.68	55.20	90.32	26.02	75.88
Calculated weighted-mean per mother cost estimate for deliveries for high-risk (complicated) pregnancies (non-Cesarean and Cesarean-Section procedures)^b^ (2015 USD)	**25.37**	**40.10**	**57.49**	**24.88**	**65.15**

^a^Delivery location included a small number of missing observations from overall sample

^b^Non-hospital delivery costs based on facility estimate and proportion of non-cesarean section facility estimate (assumed village-level = 25% of hospital, non-hospital clinic = 50% of facility cost estimate)

All cesarean section deliveries were assumed to be conducted in hospital facility

^c^Persons with > 4 ANC visits with ultrasound assumed to have four ultrasound visits for cost calculation

Higher-risk pregnancies included multiple gestation, placenta previa, and breech presentation

^d^Cost estimates for delivery type for with and without cesarean section delivery from Table 2 (all costs 2015 USD)

**Table 5:** Sub-sample probabilities and cost estimates (2015 USD) for complication-related healthcare use

	DRC	Kenya	Zambia	Guatemala	Pakistan
**Use of hospital / referral services for treatment of complications**^a^					
When did the complication occur, sub-sample of overall sample (N)	172	173	182	1351	192
During antenatal care (%)	42.10	22.00	31.90	27.00	2.70
At time of delivery (%)	49.6	69.90	65.70	68.50	95.40
Post delivery (%)	8.40	8.10	2.50	4.50	2.00
Was mother referred for a treatment of a serious pregnancy complication, (N)	172	173	182	1351	192
Yes (%)	92.70	79.15	89.15	96.95	77.10
No (%)	7.30	20.85	10.85	3.05	22.90
Did mother go to the referral facility, (N)	150	137	163	1309	147
Yes (%)	88.50	97.75	98.65	97.50	100.00
No (%)	11.50	2.25	1.35	2.50	0.00
What type of provider did mother see during visit (checked all that applied), (N)	141	134	160	1276	147
OBGYN (%)	0.60	22.55	50.10	24.55	36.70
Medical Officer (%)	37.55	27.20	29.60	76.05	29.30
Clinical Officer/Medical Assistant (%)	3.95	21.80	2.35	0.95	0.70
Nurse (%)	76.05	61.95	81.45	92.80	13.60
Sonographer (%)	7.40	7.05	5.10	0.10	0.70
Other (%)	2.10	0.00	0.00	0.25	19.75
Where was referral care received (checked all that applied), (N)	141	134	160	1276	147
Hospital Wards (%)	0.60	12.45	0.45	10.85	15.65
Intensive Care Unit (%)	0.00	0.85	0.00	0.80	0.00
Casualty (%)	0.85	0.00	0.00	88.55	0.00
Outpatient Facility (%)	0.00	11.85	0.95	1.05	1.40
Maternity Ward (%)	65.10	73.50	98.60	6.20	43.50
Other (%)	33.45	1.35	0.00	0.75	40.80
Was mother admitted, N (%)	141	134	160	1276	147
Yes	87.30	85.10	91.95	100.00	89.10
No	12.70	14.90	8.05	0.00	10.90
**Select maternal healthcare resource use (sub-sample collected in countries)**^b^					
*Maternal healthcare resource use (N)*	145	142	163	1282	147
Antibiotics (% yes)	86.25	78.70	43.95	83.30	93.90
Estimated cost per antibiotic treatment (2015 USD)	7.00	36.97	3.31	12.00	9.58
Hysterectomy after complication (% yes)	2.85	0.65	0.00	0.90	0.00
Estimated cost per hysterectomy treatment (2015 USD)	27.14	214.24	26.49	155.96	171.87
Estimates cost hospital stay for hysterectomy (2015 USD)	27.14	214.24	26.49	155.96	171.87
Other surgery (% yes)	2.85	2.75%	0.45	11.30	2.05
Estimated cost per non-hysterectomy surgery (2015 USD)	32.56	214.24	90.88	155.96	82.50
Estimates cost hospital stay for other surgery (2015 USD)	32.56	214.24	90.88	155.96	82.50
Anesthesia (% yes)	17.50	30.35	8.40	61.35	2.75
Estimated cost per use of anesthesia (if available) (2015 USD)	143.82	10.08	0.00	20.58	0.00
Blood transfusion (% yes)	13.25	6.10	3.20	4.50	21.70
Estimated cost per blood transfusion (if available) (2015 USD)	9.95	11.76	0.00	47.84	19.48
Inpatient ultrasound (% yes)	9.50	11.65	8.20	78.20	27.90
Estimated cost per inpatient ultrasound (2015 USD)	16.28	10.64	13.25	5.50	4.30
Chest radiograph (% yes)	2.00	0.0	0.95	1.80	1.40
Estimated cost per chest radiograph (2015 USD)	13.76	8.07	5.30	9.70	2.50
**Estimated mean facility-level per person added cost estimate for maternal complication events (2015 USD), for typical cohort of patients**	**37.75**	**48.68**	**3.41**	**67.31**	**17.83**
**Select neonatal healthcare resource use (sample ranges in countries per use type, N)**^c^	4660-4908	7493-9288	6363-9456	15,827-16,394	3544-7284
Neonatal hospitalization (% yes)	0.15	0.75	0.50	3.35	2.40
Estimated per person cost for neonatal hospitalization (2015 USD)	5.44	2.90	46.30	77.61	73.31
Neonatal bath within six hours after delivery (% yes)	0.10	27.85	0.80	36.80	13.30
Estimated per person cost for neonatal bath (2015 USD)	0.00	0.00	1.32	1.31	0.00
Neonatal antibiotics (% yes)	1.55	0.50	0.20	3.80	10.80
Estimated per person cost for antibiotics (2015 USD)	7.00	36.97	3.31	12.00	9.58
Neonatal oxygen use (% yes)	0.00	0.80	0.35	4.30	12.05
Estimated per person cost of oxygen (2015 USD)	29.31	1.01	1.85	6.16	4.66
Mechanical ventilation (% yes)	0.40	0.45	0.15	0.55	0.20
Estimated per person cost for ventilation (2015 USD)	0.00	0.00	1.32	39.58	4.66
Medical eye care (% yes)	1.25	15.20	1.00	56.25	0.90
Estimated per person cost for eye care (2015 USD)	0.71	0.20	1.32	4.72	0.00
Medicinal cord care (% yes)	2.65	2.00	1.40	99.30	2.30
Estimated per person cost for cord care (2015 USD)	0.00	0.00	1.21	0.66	0.00
Vitamin K (% Yes)	0.10	1.15	0.45	56.85	3.40
Estimated cost per vitamin K treatment (2015 USD)	1.90	2.02	0.74	1.31	0.58
**Estimated mean facility-level per person added cost estimate for neonatal complication events (2015 USD), for typical cohort of patients**	**0.13**	**0.27**	**0.22**	**8.15**	**0.03**
**Combined estimated mean facility-level per person added cost estimate for maternal and neonatal complication-related events (2015 USD), for typical cohort of patients**	**37.88**	**48.94**	**3.63**	**75.46**	**17.87**

^a^Percentages for use of hospital services due to complications during pregnancy and percent of complications reported are based on non-weighted average reported in cohort sub-samples in countries

^b^Probability and cost estimates presented in the lower portion of Table 5 are taken from Table 2 (unit costs) and Table 3 (complication probabilities), and are not based on the event probabilities in the upper portion of Table 5

Samples were from different data sources for probabilities for maternal health major procedures/treatments, and neonatal events

If item-level or event-level facility cost estimate $0, NA, or missing from Table 3, $0 estimate used for calculation in Table 5

^c^Exact sample size of neonatal healthcare use varied slightly per event (range is presented per country)

Components of combined estimates for maternal and neonatal costs may not reflect rounding precision given USD two decimal standard

Table 4 presents estimates of per person average costs for ANC outpatient visits (without and with US), as well as for a weighted average of combined non-US visits and antenatal screening US visits, roughly mimicking what would be expected in a typical patient cohort if antenatal screening US was used. The average number of overall ANC visits (with or without US) by country ranged from 3.20–4.45. If all ANC visits were assumed to be without US, average costs were estimated to be 15─18 USD in all countries. Total average costs for outpatient antenatal screening US ranged from 7 USD to 24 USD in countries, based on a weighted average of the reported frequency of US visits, primarily from the CRCT intervention arm. The average number of visits with antenatal screening US ranged from 1.53─1.97 among countries. Patients generally received one-three scans and a small portion received four or more antenatal screening US scans. For a combined expected total ANC cost for visits (non-US and antenatal screening US visits), estimates ranged from 15─16 USD in Pakistan and Guatemala to 25─31 USD in Kenya, DRC, and Zambia. In general, antenatal screening US visits increased the overall estimate for ANC visit costs, as a function of the unit cost estimate for the antenatal screening US visits within countries and the frequency of US visits. For example, antenatal screening US visits in DRC were estimated to cost more than double non-US ANC visits, while in Guatemala and Pakistan, antenatal screening US visit costs were estimated to be closer to ANC visits without US.

Table 4 also presents estimates of delivery costs based on the frequency of alternative birthing locations/settings (local, clinic, and hospital). Per mother average delivery costs ranged from 23 USD in DRC and Guatemala to 58 USD in Pakistan (31 USD in Kenya and 52 USD in Zambia) for the overall sample available from the CRCT and GN MNHR patients, based on reported delivery location and type of delivery. Costs associated with cesarean section deliveries were included based on the percentage of these deliveries reported. Delivery cost estimates for high-risk pregnancies delivering in the three settings are presented separately in Table 4. These sub-sample pregnancies resulted in higher costs in all countries (roughly 10–28% greater per country) due to higher cesarean section rates in the higher-risk group compared to the overall sample. Those having cesarean section deliveries were the same mothers for both the overall sample and sub-sample. Guatemala had the highest rates of cesarean section deliveries (28% in the overall sample and 34% in high-risk pregnancies). Other countries ranged from 1 to 11% cesarean section rates in the overall sample, and 3 to 15% for the higher-risk, more complicated pregnancy group.

### Maternal and neonatal complications resulting in additional healthcare use

Table 5 presents data from a sub-sample collected in each country during the CRCT, showing when complications occurred during pregnancy and what types of services and locations were reported for healthcare services for mothers. The majority of pregnancy-related complications in the sub-sample occurred at the time of delivery or post-delivery, accounting for roughly 50–70% of complications, with a greater portion occurring at delivery in Pakistan (95%). Table 5 also presents data from two sub-samples to estimate costs associated with maternal complication and neonatal complication events. Based on the probability and cost estimates reported, an additional 4─75 USD is estimated to be expended by a health facility to manage select maternal and neonatal complications in a cohort similar to our CRCT and GN MNHR. Cost estimates in Zambia were particularly low relative to per capita income level, due to some cost parameters not being available (zero-dollar estimates) or included as part of bundled, episode-based delivery-related reimbursement amounts. Another factor influencing complication-related costs across countries was variable frequencies reported of certain complications in our sub-samples. Limited testing of ranges of costs for resources in Zambia with a zero USD estimate does not greatly increase overall costs in Zambia or in other countries, due to the overall low frequency of these events. Complication management cost estimates were highest on average in Guatemala, influenced by a higher frequency of reported neonatal events (Table 5) compared to other countries, and that most item-level cost estimates were non-zero.

**Fig. 1:**
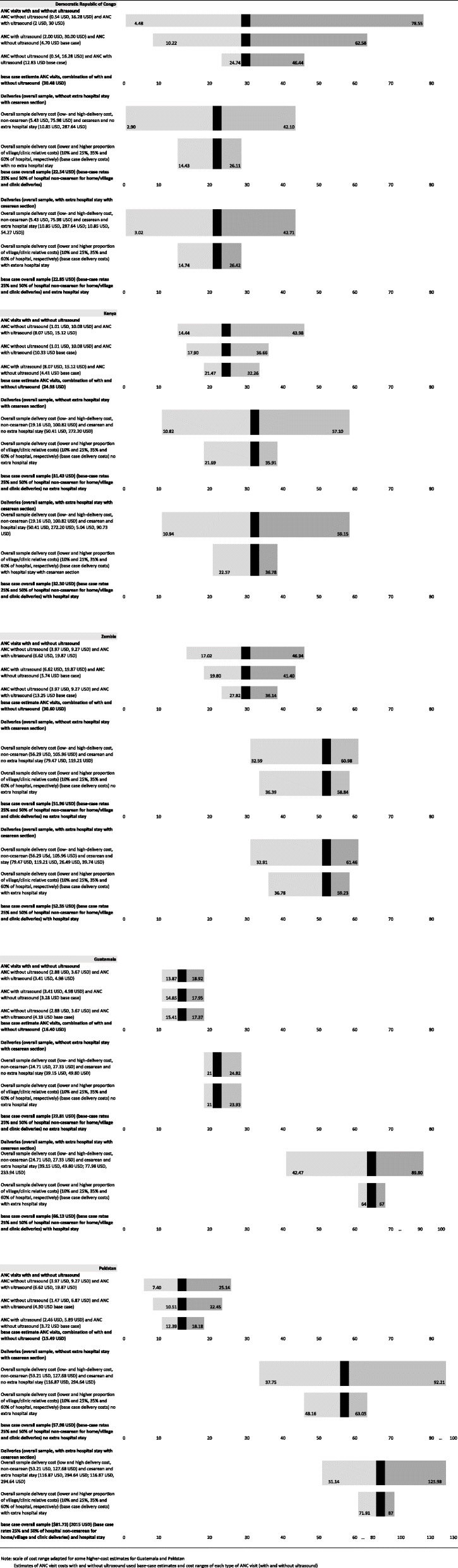
Two-way and one-way sensitivity of ANC visits and deliveries (2015 USD). Note: scale of cost range adapted for some higher-cost estimates for Guatemala and Pakistan Estimates of ANC visit costs with and without ultrasound used base-case estimates and cost ranges of each type of ANC visit (with and without ultrasound)

Figure 1 presents sensitivity testing of cost estimates in Table 4 for ANC costs and delivery costs. Some countries had tighter ranges of low and high unit cost estimates for ANC visits, e.g., Guatemala and Pakistan, and thus had smaller changes in these base-case costs due to adjusting parameter values. Other countries, e.g., DRC, exhibited more variability in ANC visit cost ranges and thus estimated average cost ranges were greater in sensitivity testing. Low- and high-range estimates in DRC were particularly large for visits with antenatal screening US. For example, combined visits with and without antenatal screening US in DRC ranged from 4─79 USD, due to a greater estimated range in US costs (high of 30 USD per visit). Delivery cost estimates in DRC, on the other hand, were lower than in other countries, but close to Guatemalan delivery cost estimates. Delivery costs in Pakistan demonstrated a higher level of variability without and with inclusion of a prolonged hospital stay associated with cesarean section deliveries (37─92 USD and 51─126 USD, respectively) compared with a base-case average of 58 USD and 82 USD, respectively (Fig. 1). These estimates were influenced by average cesarean and non-cesarean delivery-based unit costs being relatively high in Pakistan compared to other countries, as well as the second highest cesarean section rates among countries. Higher delivery costs in Guatemala were influenced by having the highest rates of cesarean sections, not higher average unit cost estimates for deliveries. In all countries, adjusting the proportional cost for local/village and clinic-level deliveries relative to hospital facility delivery costs had less influence on average base-case estimates compared to using the low- and high-end of the delivery-related unit cost estimates. Due to lower rates of cesarean section deliveries in the three African countries compared to Guatemala and Pakistan, including additional hospital stays associated with cesarean deliveries had minimal impact on overall delivery cost estimates.

## Discussion

We estimated average costs for ANC-related service use and delivery-related services, as well as for select complication events in five LMICs in different global regions: DRC, Kenya, Zambia, Guatemala, and Pakistan. Our economic study was part of a large CRCT and ongoing maternal health registry to support efforts to better document resource use and costs associated with antenatal screening US and other healthcare services [8]. We estimated a broad range of ANC-related resource use from participating countries in order to inform global health researchers and health policy-making stakeholders, as well as to be available as inputs in subsequent economic assessments related to these LMICs.

Our initial goal was to determine if routine antenatal screening US was economically efficient due to lower costs overall to provide screening, even if other outcomes were similar. For this type of economic study, we would often use a “societal perspective” that looks at all direct medical and indirect costs in “opportunity cost” terms from multiple perspectives, and compare costs with effectiveness outcomes. However, in terms of the CRCT’s clinical effectiveness evidence for the five countries combined, and in individual countries, the primary composite endpoint of maternal mortality, near-miss maternal mortality, stillbirth, and neonatal mortality did not differ significantly between the US intervention arm (*n* = 24,008 pregnant women) and the control arm (*n* = 22,896 pregnant women). Process-related outcomes, such as the mean number of ANC visits, delivery location, cesarean section delivery rates, complicated deliveries, or six-week post-partum hemorrhage events, were also not statistically different between CRCT arms [12]. Since effectiveness in all countries was so similar, cost-effectiveness assessment was not a practical approach to compare groups. Therefore, we estimated unit costs for particular ANC and delivery-related resource use, and constructed key event average cost estimates from these unit costs within each country, based on events from the CRCT and GN MNHR [12, 13]. We report cost assessments for combined treatment groups in countries based on estimates of average costs of ANC visits (with and without antenatal screening US), delivery events, and the management of select complications.

In this economic study conducted alongside the CRCT, we were able to successfully estimate average costs associated with resources required for most ANC procedures and services in participating countries, using a facility perspective. Estimates were primarily based on reimbursement schedules and expected payment amounts when available. Our local country experts obtained hospital or facility administration assistance when “prices” were not readily available publicly. Overall, the antenatal screening US group was essentially dominated by standard of care, with higher ANC visit costs and similar effectiveness outcomes. There is more time and higher expected average unit costs associated with antenatal screening US visits compared to non-US ANC visits. We did not observe differences in the frequency of hospital deliveries or cesarean section deliveries between CRCT groups in countries, as we initially expected. Antenatal screening US was expected to increase hospital costs, through improving the planning of deliveries for higher-risk pregnancies. Hospital events and thus expected average hospital costs were not different between randomized groups. However, for US, there would be additional costs associated with acquiring US equipment, training sonographers, and maintaining portable US equipment. Estimating these costs was beyond our study’s scope.

To put our findings in context, it helps to recognize a few key country-level characteristics that influence the interpretation of findings and suggest further research is needed in these countries representing different global regions. The five LMICs in the CRCT are diverse in terms of gross domestic product per capita, health spending per capita, maternal and neonatal mortality rates, and use of US overall. Countries had differences in basic infrastructure, health system access or readiness, and insurance coverage policies. Our primary focus was on public facilities serving the largest portions of populations in participating countries. Despite select differences in cost estimates for resource use among countries, absolute and relative costs associated with antenatal care services were generally aligned with per capita income levels.

Each country included publicly provided healthcare services, although Pakistan and Guatemala also have a greater prevalence of private health sector facilities and services, including the provision of US services. Although we did not have a formal evaluation of the quality of private sector-provided US services, used by some in the control groups, our clinical team was able to verify the quality of sonographer training and the antenatal screening US exams performed in the intervention clusters, to ensure sonographers were meeting training requirements and that US exams met appropriate clinical standards. More advanced equipment, services, or specialty healthcare personnel were not available in some countries or in some facilities, making precise estimation challenging. Therefore, our unit cost estimates sometimes had large low-to-high ranges. We used a country-level average for all facilities within countries, if a particular service was available at least one facility. Our ANC use and delivery-related cost estimates provide extensive unit cost data and event-related cost estimates for the five participating countries. We also tested sensitivity ranges related to average costs for ANC visits and delivery events using different unit cost scenarios.

We acknowledge the importance of having appropriately trained sonographers for our screening study. In terms of training the sonographers in the CRCT who conducted the antenatal screening ultrasound exams, we developed an extensive training curriculum that is published elsewhere [10, 20–23]. There is a severe shortage of trained sonographers serving rural communities in LMICs, including the five countries that participated in the CRCT. It would not have been possible to recruit fully trained sonographers to staff all of the 25 intervention clusters in the study. A secondary objective of the study was to evaluate whether US naïve healthcare workers (midwives, clinical officers, nurses, radiographers) could be trained in a short, intensive course followed by a period of supervised scanning to provide accurate screening ultrasound. The concept was not to train independent sonographers but rather to train extenders of the fully trained sonographers located at designated referral facilities. Any patient with a suspected abnormality identified at an intervention cluster health center would be referred to the referral hospital for confirmation by a fully trained sonographer.

There were similarities in health system structures in some countries, such as Kenya and Zambia, and numerous differences in others, such as with the DRC, Pakistan, and Guatemala. Since most participating hospitals in the CRCT were rural, resource availability was more limited in these facilities compared to urban hospitals or teaching hospitals in countries. Particularly in rural areas, the lack of public transportation infrastructure and long travel distances contribute to challenges in patients accessing health clinics and hospitals. The Kenyan and Zambian health systems largely consist of public facilities providing services, as well as basic public healthcare insurance coverage and government payments to facilities for delivery services, with some private centers and private insurance options used mostly by employees of larger companies. In contrast, in the DRC there is essentially no public health insurance coverage and patients typically pay “out-of-pocket” or make other arrangements with a health center to receive services. Guatemala and Pakistan have a mixture of public and private sector healthcare provision, with urban areas having more private clinics, partly related to higher-income levels and market structures. In addition, several countries rely on external financial donors and non-governmental organizations (NGOs) to subsidize healthcare and/or infrastructure spending, influencing short-term use and cost scenarios as well as creating dependence or constraints on longer-term sustainability.

The DRC, the country with the lowest per capita income level, had a synchronous complicating factor independent of the trial. Hospital care was subsidized by an international non-governmental organization, lowering the financial burden on families. However, the longer-term sustainability of these types of initiatives are uncertain and may reduce the use of hospital services in the absence of such subsidies. Hospital and clinic resources in the DRC are limited [24–26] and our individual cost estimates may represent higher costs than feasible for families to afford without subsidization. As our sensitivity analysis showed, base-case cost estimates in the DRC varied widely based on unit cost estimates for visits and deliveries. As expected, DRC had fewer hospital deliveries and cesarean section deliveries compared to other countries, with low rates similar to Kenya and Zambia. Since most deliveries in these three countries occurred in non-hospital clinic settings or in the home/village, lowering the assumed cost of village-level or clinic-level deliveries, as a proportion of hospital delivery costs, had the effect of lowering expected overall average delivery costs.

Since US equipment can be used to assess many patients during its life cycle, the average per patient or per event usage cost is expected to be relatively low when allocated among thousands of patients during each of multiple years. However, investment and maintenance costs, for equipment and sonographer training, and for subsequently higher sonographer wages, may be prohibitive in LMICs. Including stakeholder (e.g., health facility) investments in ultrasound equipment would add costs to our estimates of care-related costs presented in the current paper. Although antenatal screening US does not appear to result in more efficient or lower cost outcomes, we are not able to claim that portable US is not efficient overall or for specific indications in a clinic or hospital setting. Some studies have shown a potential clinical utility of US in assessing gynecologic, abdominal, musculoskeletal, cardiovascular, pulmonary, traumatic, and other conditions [27]. Estimating secondary effects of having portable US available in clinics and hospitals was outside the scope of our assessment. For ultrasound equipment purchases, facilities and other stakeholders would have to estimate benefits and costs of their overall investment, including estimating expected return on investment considering all potential uses of ultrasound.

Participating clusters at each country site had designated referral hospitals where antenatal screening ultrasound findings identified at the rural health centers were confirmed by fully-trained sonographers. The details of each country site referral process varied somewhat, but when a referral was required from a remote health center, the hospital sonographer was texted with the patient name and provisional diagnosis. The patient was given a report from the health center along with key antenatal screening ultrasound images to be given to the hospital sonographer. The importance of attending the referral was explained to the patient. Not surprisingly, the success of the referral process varied somewhat from health center to health center and from country to country. Follow-up and referral indications were consistently performed by a “referral algorithm” at each health center.

Our resource use and cost identification study included several limitations influencing data collection and interpretation. Despite efforts to maintain consistency in data collection among countries, we collected cost information from a variety of sources to estimate average antenatal care and delivery-related costs. We did not specifically estimate the cost of patient referrals for elevated care during the study. The proportion of patients receiving referrals were not statistically different between the randomized groups in any country. We did not assess all health centers within countries, and surveyed a sampling of representative centers. Different countries had varying amounts of available data for services and prices, e.g., reimbursement schedules. We estimated outpatient and inpatient service costs for variables of interest, although specific services or equipment were not available in some countries and/or in some facilities, or were part of bundled reimbursement for deliveries. Thus, we could not estimate unit costs for all items but we did not impute values for missing cost estimates. We estimated costs based on the average number of ANC visits, delivery locations, complication-related use reported in the CRCT or GN MNHR, rather than using retrospective claims data or tracking hospitalizations for patients to assess use. With more than 45,000 deliveries and approximately 160,000 antenatal visits during the CRCT, the cost of collecting resource use on each of these was prohibitively expensive. We acknowledge that reimbursement amounts may not represent the cost of service provision but only serve as proxies for costs, particularly for multi-use equipment and multi-function personnel. We also did not assess indirect costs, such as time costs or productivity costs. Routine antenatal screening ultrasound is likely to add to patient-level costs, without expected effectiveness gains.

## Conclusion

Despite substantial differences among countries in infrastructures and health system capacity, there were similarities in resource allocation, delivery location, and country-level challenges for providing high-quality ANC. Overall, there was no clear suggestion that adding antenatal screening US would result in either major cost savings or major cost increases. However, antenatal screening US would have higher training and maintenance costs. Given the lack of clinical effectiveness evidence and greater resource constraints of LMICs, it is unlikely that introducing antenatal screening US would be economically efficient in these settings—-on the demand side (i.e., patients) or supply side (i.e., healthcare providers).

## Supplementary Information

**Additional file 1: Supplementary Figure 1.** Cluster Randomized Controlled Trial CONSORT Diagram.

## Data Availability

The datasets used and/or analyzed during the current study are available from the corresponding author on reasonable request.

## Abbreviations

ANC: Antenatal care
CRCT: Cluster randomized controlled trial
GN MNHR: Global Network Maternal Newborn Health Registry
LMICs: Low- and middle-income countries
US: Ultrasound
USD: United States dollars
