# Supplementary figures and images for: Cost estimation alongside a multi-regional, multi-country randomized trial of antenatal ultrasound in five low-and-middle-income countries
Source: BMC Public Health. 2021 May 20;21:952. doi: 10.1186/s12889-021-10750-8 (PMC8135981; doi:10.1186/s12889-021-10750-8)

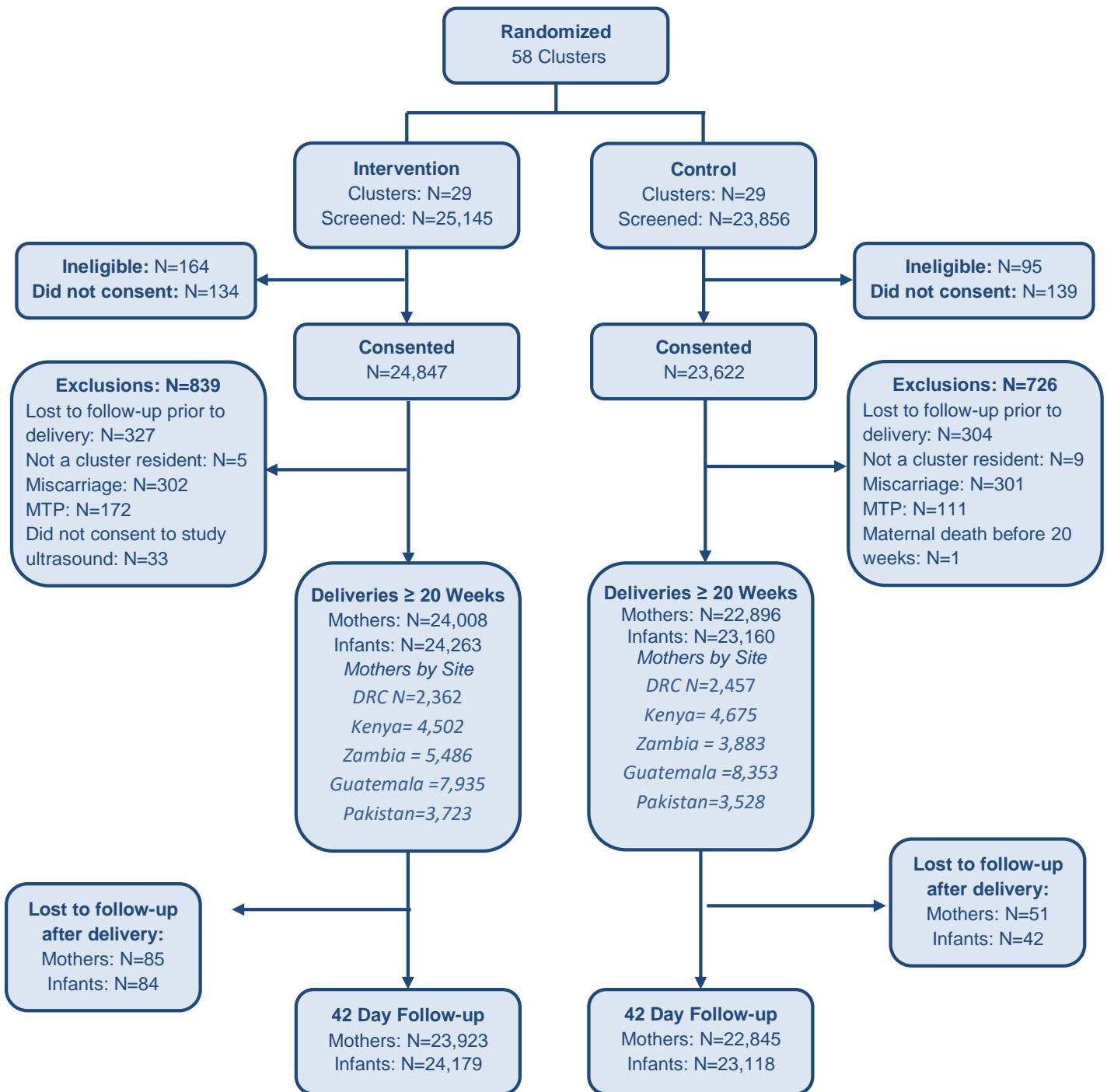

**Figure S1.** First Look Trial CONSORT Diagram.

Supplement: Supplementary file 1 — Additional file 1: Supplementary Figure 1. Cluster Randomized Controlled Trial CONSORT Diagram. [file 12889_2021_10750_MOESM1_ESM.pdf]
